# Identifying the Representational Structure of Affect Using fMRI

**DOI:** 10.1007/s42761-020-00007-9

**Published:** 2020-04-18

**Authors:** Alison M. Mattek, Daisy A. Burr, Jin Shin, Cady L. Whicker, M. Justin Kim

**Affiliations:** 1 Department of Psychology, University of Oregon, Eugene, USA; 2 Department of Psychology & Neuroscience, Duke University, Durham, USA; 3 Department of Psychological & Brain Sciences, Washington University in St. Louis, St. Louis, USA; 4 Department of Psychiatry & Behavioral Sciences, Stanford University, Stanford, USA; 5 Department of Psychology, Sungkyunkwan University, Seoul, South Korea

**Keywords:** Affect, Emotion, Valence, Arousal, Amygdala

## Abstract

The events we experience day to day can be described in terms of their affective quality: some are rewarding, others are upsetting, and still others are inconsequential. These natural distinctions reflect an underlying representational structure used to classify affective quality. In affective psychology, many experiments model this representational structure with two dimensions, using either the dimensions of valence and arousal, or alternatively, the dimensions of positivity and negativity. Using fMRI, we show that it is optimal to use all four dimensions to examine the data. Our findings include (1) a gradient representation of valence that is anatomically organized along the fusiform gyrus and (2) distinct sub-regions within bilateral amygdala that track arousal versus negativity. Importantly, these results would have remained concealed had either of the commonly used 2-dimensional approaches been adopted a priori, demonstrating the utility of our approach.

The events we experience can be distinguished in terms of how they affect us in a very broad sense: we can evaluate whether an event is qualitatively rewarding or aversive (positive/negative) or whether it is relatively inconsequential (neutral). The *arousal* dimension distinguishes neutral from rewarding/aversive events, and the *valence* dimension distinguishes rewarding from aversive events. Affective quality, which can be assigned to any event or experience, allows us to predict a wide range of behavioral response patterns, including which events will be perceived, attended to, remembered, approached, or avoided. Due to this predictive power, psychologists and neuroscientists alike have shown great interest in how the dimensions of affective quality are represented in the brain (e.g., Chikazoe, Lee, Kriegeskorte, & Anderson, 2014; Kim et al., 2017; Lindquist, Satpute, Wager, Weber, & Barrett, 2015; O'Doherty, Kringelbach, Rolls, Hornak, & Andrews, 2001; Fox, Lapate, Shackman, & Davidson, 2018; Shackman & Wager, 2019). This report examines how the perception of affective quality is related to neural activity as measured by fMRI.

The principal dimensions of affect (valence and arousal) are so ubiquitous that they permeate numerous domains of psychology and other related fields, such as economics (where decisions are influenced by positively-valenced economic gains and negatively-valenced losses), learning and reinforcement theory (where behavior is determined by positively-valenced rewards or negatively-valenced punishments), and even clinical theory (where diagnostic categories can be organized by positive-valence or negative-valence symptom profiles; e.g., Hariri, 2015). Across all of these subfields, researchers often characterize brain responses (or other physiological or behavioral responses) in terms of what happens following an affectively-charged event. The unseen challenge in this domain, however, is that any experiment will necessarily have to make theoretical assumptions about the affective dimensions themselves. These assumptions will inherently scaffold any experimental design and/or model that is meant to investigate some particular behavioral or physiological response associated with the affective quality. The relative appropriateness of the theoretical assumptions will, in turn, constrain the nature of the experimental results. Although investigations about affective quality are numerous, to date, the choice of which theoretical assumptions to impose on the affective dimensions remains an experimenter degree-of-freedom, as there has been considerable debate about the ontological structure of valence and arousal (for reviews, see Mattek, Wolford, & Whalen, 2017 and Brainerd, 2018). Moreover, researchers do not generally motivate or describe which theoretical structure they are subscribing to, even though this choice is inextricably tied to the nature and interpretation of their results.

## Background

**Fig. 1:**
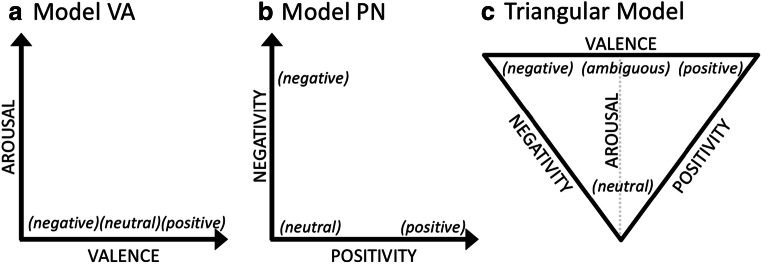
Affective quality is routinely represented in one of two ways: **a** using the dimensions of valence and arousal (Model Valence Arousal) or **b** using the dimensions of positivity and negativity (Model Positivity Negativity). **c** An alternative triangular model (from Mattek et al., 2017) that allows the assumptions in both columns 1 and 2 to be true under certain conditions, rather than logically opposed as they appear in **a** versus **b**

There are two established theories about affective dimensions that have gained significant traction in experimental work, and their premises are logically opposed to each other (see Table 1 for a depiction of this logical opposition). One approach (referred to as Model Valence Arousal), which can be represented with a Cartesian plane that has valence of the *x*-axis and arousal on the *y*-axis (Fig. 1a), posits that (a) valence is best represented with a line (i.e., the degree to which an event is positive *can be predicted* by inverting the degree to which it is negative; e.g., Russell, 2017) and that (b) changes in arousal *cannot be predicted* by changes in valence (i.e., changes in arousal happen independently from changes in valence; e.g., Russell, 1980). An alternative approach (referred to as Model Positivity Negativity), which can be represented with a Cartesian plane that has positivity on the *x*-axis and negativity on the *y*-axis (Fig. 1b) posits that (a) valence is best represented with a plane or two orthogonal lines (i.e., the degree to which an event is positive *cannot be predicted* by considering the degree to which it is negative; e.g., Cacioppo & Berntson, 1994) and that (b) changes in arousal *can be predicted* by changes in valence (i.e., arousal is a linear combination of the two valence dimensions; e.g., Watson & Tellegen, 1985; Lang, 1995; Kron, Pilkiw, Banaei, Goldstein, & Anderson, 2015).

**Table 1:** Illustration of the logical opposition between the existing approaches for measuring and modeling dimensions of affective quality. VA = Valence Arousal; PN = Positivity Negativity

	Model	
	VA	PN
Can positivity be predicted by negativity?	Yes	No
Can arousal be predicted by valence?	No	Yes

Note: 1. The green-principled group is the basis of comparison with the other groups and is not shown on the table

Despite the logical opposition of these two premises, both sets of assumptions are supported by large bodies of observed data, resulting in researchers having to arbitrarily choose which set of assumptions to adopt for any given experiment. Conveniently, a newly proposed synthesis of these two theories has demonstrated how to predict which of these two sets of assumptions will be supported by observed rating data. Specifically, this higher-order prediction can be made by considering a third variable, *valence ambiguity*, which reflects the consistency with which an event is assigned a particular valence value (Mattek et al., 2017; Brainerd, 2018). That is, when ambiguity is high, the first set of assumptions (Model Valence Arousal) will be correct, but when it is low, the alternative set of assumptions (Model Positivity Negativity) will be correct, and this newly proposed theoretical principle has been mathematically formalized with a set of equations (Mattek et al., 2017). Importantly, this new theory emphasizes that valence, arousal, positivity, and negativity are all partially independent. That is, all four dimensions can be considered in experimental work moving forward.

In this paper, we use an fMRI experiment to illustrate how critical this theoretical issue is when it comes to the interpretation of experimental data. Here, we find that the nature of the experimental results is completely contingent of whether one adopts Model Valence Arousal versus Model Positivity Negativity to approach the data. Moreover, the synthesis of the two sets of results yields interpretable anatomical arrangements of brain activity, which supports the utility of theoretically synthesizing the existing approaches, as is done in Mattek et al. (2017).

## Methods

### General Approach and Experimental Predictions

**Fig. 2:**
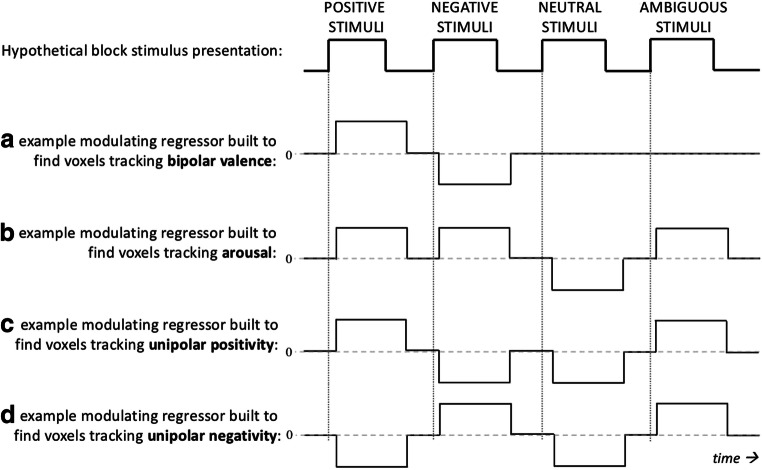
A cartoon example of how different regressors, and hence different assumptions about voxel responses, are employed depending on which theoretical approach is chosen to analyses the same data. The hemodynamic response is not considered here, and the choice of hemodynamic-response parameters is beyond the scope of this paper. Also note that this figure assumes that the conditions positive, negative, and ambiguous all have the same arousal value, but the height of these regressors would normally vary according to continuous values on the respective measurement scales. Nonetheless, this figure illustrates how each affect theory looks for substantially different voxel response shapes. Consequently, choosing one theory or the other is the current status quo in the field of affective science, despite the fact that there is no a priori evidence that voxel responses fit any of these regressors better than the others

How can these theoretical approaches be investigated with fMRI? First, consider the measured activity of any given voxel in the brain,^1^ which could potentially exhibit activity variation that is best predicted by either (a) *a valence contrast* that compares positive versus negative conditions (with ambiguous and neutral conditions set to zero), which would be modeled with a regressor such as the one shown in Fig. 2A; (b) *an arousal contrast* that compares higher arousal conditions (positive, negative, and ambiguous) to lower arousal conditions (neutral), which would be modeled with a regressor such as the one shown in Fig. 2B; (c) *a positivity contrast* that compares conditions with positivity (positive and ambiguous) versus conditions with no positivity (negative and neutral), which would be modeled with a regressor such as the one shown in Fig. 2C, or (d) *a negativity contrast* that compares conditions with negativity (negative and ambiguous) versus conditions with no negativity (positive and neutral), which would be modeled as a regressor such as the one shown in Fig. 2D. To adopt any of the theoretical assumptions listed in the previous section inherently involves making a priori predictions about how these contrasts will fit the measured activity, which are described in the next few paragraphs.

To begin, if we adopt Model Valence Arousal and assume that positivity and negativity are opposites (i.e., valence is linear), this makes an a priori prediction that the measured responses should differ proportionally to the difference between positive and negative conditions. That is, the assumption of linear valence predicts that comparing a positive condition to a negative condition (valence contrast) will maximize the effect of valence, whereas comparing a positive condition to a neutral condition (or a negative to a neutral condition; i.e., positivity or negativity contrasts) would result in a weaker valence effect. That is, if the voxel activity actually looks more like Fig. 2A, then that regressor is a better choice than the regressors in Figs. 2 B and C. For many studies that look at valence, the regressor in Fig. 2A would be used, and those in Fig. 2 B and C would not even be tested, because the assumption that valence is bipolar takes for granted that Fig. 2A will work better than Fig. 2B or Fig. 2C, even though this is generally not explicitly tested. For fMRI, the assumption of linear valence also inherently predicts that the coefficients from a negativity contrast (as in Fig. 2C) will have opposite signs compared with a positivity contrast (as in Fig. 2D) and that these effects will occur in overlapping voxels. That is, if only one bipolar regressor were used to model valence, there is no possibility of splitting apart effects of positivity and negativity, even if they do exist as separate effects in the data.

On the other hand, if we adopt Model Positivity Negativity and assume valence is non-linear (i.e., if we assume valence is at least two dimensions and brain activity in a positive condition is not the opposite of brain activity in a negative condition), then regional activity during a positive condition will be equally different from a neutral and a negative condition. In this case, the underlying prediction that comes along with the assumption of non-linear valence is that positivity and negativity contrasts will yield stronger effects compared with a valence contrast. That is, if voxel activity actually looks like Fig. 2B or Fig. 2C, as is assumed by Model Positivity Negativity, then a biopolar valence regressor like the one in Fig. 2A would not fit the data as well. Using two separate regressors for positivity and negativity also allows the effects of positivity to be in anatomically distinct brain regions compared with the effects of negativity.

Finally, there is an assumption that changes in arousal can be predicted from changes in positivity and negativity that is inherent to some versions of Model Positivity Negativity. This assumption would predict observed anatomical overlap between an arousal contrast compared with a positivity contrast and a negativity contrast. Specifically, if it is correct to assume Model Positivity Negativity, regional activity that is linearly proportional to an arousal contrast should be the union of regions that are linearly proportional to positivity and negativity contrasts, given that the regressor in Fig. 2D is the sum of the regressors in Figs. 2 B and C. However, if changes in arousal are at least partially independent from changes in valence, regional activity proportional to arousal should be in anatomically distinct voxels compared with the regions proportional to positivity and/or negativity.

These predictions lay out very specifically how the assumptions of established theoretical structures can be tested. Conveniently, the multivariate nature of fMRI measurements makes it more obvious how all of the assumptions, despite their logical opposition, can be simultaneously true, which is also afforded by the new, synthesized theoretical structure (Mattek et al., 2017). That is, in theory, any particular voxel might show a response pattern that is most closely aligned with linear valence, arousal, positivity, or negativity. However, in current practice, experiments that involve manipulations of affective quality generally do not examine all four dimensions, and the reason that all four dimensions are not examined in current practice is justified by the prevailing theories, which claim that some of the dimensions are redundant and therefore unnecessary (see “Background”). That is, if positivity is the opposite of negativity (Russell, 2017), there is no reason to have more than one valence contrast. On the other hand, if arousal is proportional to valence (Lang, 1995; Kron et al., 2015), there is no reason to have an arousal contrast in addition to valence. One of the major proposals offered by the new theoretical synthesis (Mattek et al., 2017) is that these dimensions are not as redundant as they are sometimes claimed to be, but also that orthogonality (lack of redundancy) cannot be assumed either.^2^ Here, we offer an experimental design and modeling strategy that effectively teases apart these partially redundant dimensions and verifies their partially independent representation in neural activity. The general design and analysis approach demonstrated here is not limited to fMRI measurements and could be applied to other physiological and/or behavioral measures to test how they are influenced by changes in affective quality.

### Participants

Thirty-two participants were recruited from Dartmouth College and the local community (*N* = 32, 19 female). This sample size has been used successfully in fMRI experiments with similar affective manipulations (e.g., Jin, Zelano, Gottfried, & Mohanty, 2015; Kim et al., 2017). In accordance with the Committee for the Protection for Human Subjects, participants provided informed consent prior to their participation and were compensated with either monetary payment or course credit following their participation. For quality control, six of these participants were excluded for excessive movement^3^ during their scan session, leaving a total of twenty-six participants (*N* = 26, 15 female, mean age = 20.1 years old). All exclusions were decided prior to group analyses in an effort to maximize the quality of the data.

### Stimuli

**Fig. 3:**
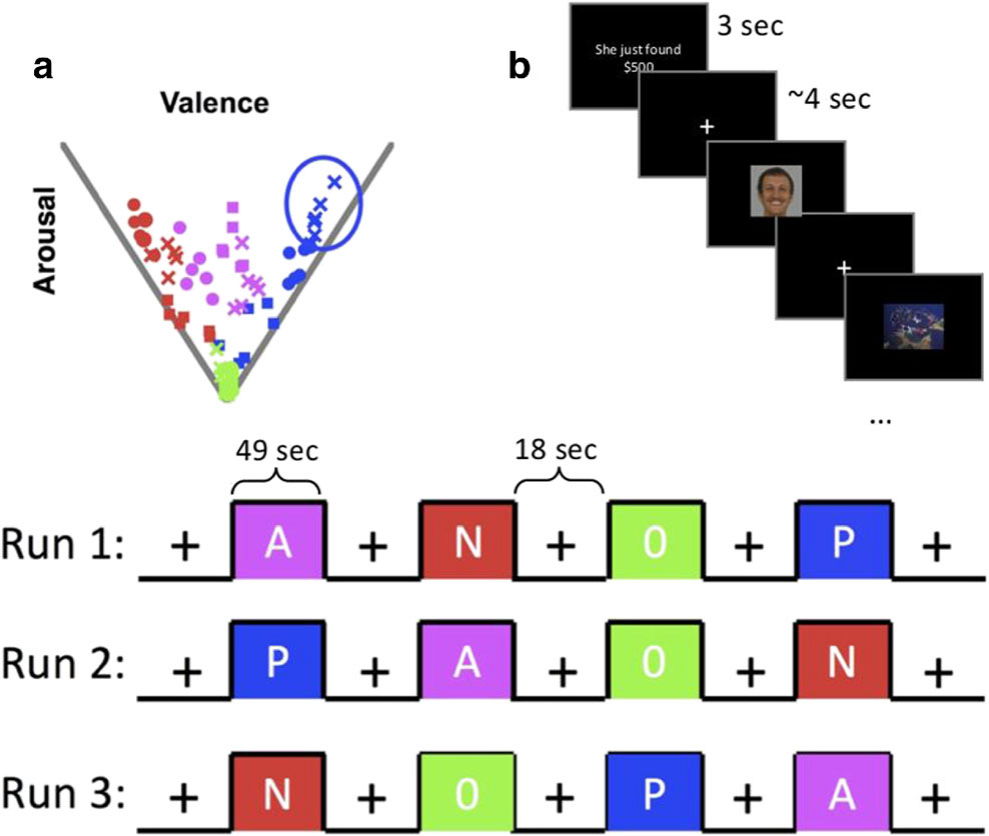
**a** The twelve clusters of stimulus items, for visualization purposes, are represented here in *valence* × *arousal* space (although they could also be plotted in *positivity* × *negativity* space and would have similar relative distances). Blue clusters represent positive stimulus items, red clusters represent negative stimulus items, magenta clusters represent ambiguously valenced stimulus items, and green clusters represent affectively neutral items. Each item is plotted according to its mean rating along each dimension, with each condition clearly occupying a different part of the space. Each color is divided into three blocks of stimuli, which are delineated by shape (cross, circle, square). Therefore, the shapes are only relevant within color and are not related to other colors of the same shape. **b** [Top] Each cluster of items contained two face items, two scene items, and two sentence items, which were presented in a pseudo-random order within a single stimulus block; [bottom] each functional run contained one block with each of the affective conditions: P = positive; N = negative; A = ambiguously valenced; 0 = affectively neutral

Experimental stimuli consisted of seventy-two items from three distinct modalities (24 faces, 24 sentences, 24 complex scenes). Faces were selected from an in-house database of emotional facial expressions, sentences were constructed based on previous work (see stimuli described in Mattek et al., 2017), and complex images were selected from either the International Affective Picture System (IAPS) or an internet search that yielded comparable images. Items were selected to span the psychological dimensions of interest, which are constrained within a triangular structure in either 2-dimensional affective space (Fig. 3a; see Mattek et al., 2017 for an in-depth discussion on this triangular structure). Note that decades of experimental work have shown that affective ratings of briefly presented stimulus items are reliably and naturally constrained within this psychological structure (e.g., see Kron et al., 2015; Knutson, Katovich, & Suri, 2014; Mattek et al., 2017), making it a suitable guideline for selecting stimuli for this experiment. In other words, stimuli outside this boundary tend to be the exception rather than the rule, and only occasionally appear in specific cultural or experimental contexts (e.g., Tsai, Knutson, & Fung, 2006; Kuppens, Tuerlinckx, Russell, & Barrett, 2013). In this sense, the triangular structure within valence and arousal space is a naturally occurring (rather than an experimentally imposed) constraint on the stimulus selection.

The stimulus items used here were organized into twelve clusters of six items each, such that each cluster sampled a localized aspect of the psychological space and contained exactly 2 faces, 2 sentences, and 2 complex scenes. The location of each item in the space was determined using data from a number of pilot experiments as well as previously published data. That is, most of the stimulus items had been rated on the dimensions of arousal, valence, positivity, and negativity in one of the eight datasets published in Mattek et al., 2017 or in similar unpublished datasets of with these ratings. A few of the items merely resembled those from previous work. For example, the most positive IAPS picture is a photo of puppies, which has been used in our previous work (e.g., Mattek et al., 2017). Instead of using that photo, which has somewhat low resolution, we used a photo of a kitten, which comparably was the most positive item in this stimulus set. In other words, we predicted that the kitten photo would be rated as having approximately the same affective quality as the puppy photo, and post-experimental ratings confirmed that this was a correct prediction on our part. Overall, post-experiment behavioral ratings demonstrated that our participants reliably categorized the stimulus items according to four affective conditions of interest: clearly positive, clearly negative, ambiguously valenced, and neutral (see section on “post-scan task”). Items within a cluster were not related to each other in any particular semantic way—the sentences did not describe the scenes or faces, rather, the only factor held constant within a cluster of stimuli was the affective quality of interest.

We tested whether low level features of the stimuli, namely luminance and sentence length, differed between conditions. On average, the luminance value was *M* = 0.502 (possible values range from 0 to 1), SD = 0.146. Using a 3 × 4 ANOVA (3 item types × 4 affective qualities), we verified that luminance was not difference according to item type (*p* = 0.435), affective quality (*p* = 0.687), or the interaction of image type and affective quality (*p* = 0.940). On average, sentence length was *M* = 5.208 words, SD = 1.414. Using a one-way ANOVA, we verified that sentence length was not significantly different between affective conditions (*p* = 0.152).

### General Procedure

After providing consent and demographic information, participants took part in a 40-min scanning session consisting of an anatomical scan, followed by six functional scans that lasted 5 min each. After the scan session, participants completed a brief computer task where they provided ratings in response to the stimuli seen in the scanner.

### Image Acquisition Parameters

All participants were scanned at the Dartmouth Brain Imaging Center using a 3 Tesla Siemens Prisma Scanner with a 32-channel head coil. Anatomical T1-weighted images were collected using a high-resolution 3D MP-RAGE sequence, with 160 contiguous 1-mm-thick slices (TE = 4.6 ms, TR = 9800 ms, FOV = 240 mm, flip angle = 8°, voxel size = 1 × 0.94 × 0.94 mm). Functional images were acquired using an echo-planar T2*-weighted imaging (EPI) sequence. Each volume consisted of 54 slices with 135 mm coverage (TE = 31 ms, TR = 2500 ms, flip angle = 79°, voxel size = 2.5 × 2.5 × 2.5 mm, PAT = 2, Grappa = 1, SMS = 2).

### fMRI Experiment Design

During each functional scan, stimulus items were ordered and timed according to a state-item design (Donaldson, Petersen, Ollinger, & Buckner, 2001) using PsychoPy software (Peirce, 2009). This design choice facilitated our ability to tease apart effects of the affective quality of the stimuli from the item modality (Somerville et al., 2012). More specifically, the twelve items from a particular localized cluster in affective space (Fig. 2A) were all presented within a single 49-s block (randomly jittered timing with a mean inter-stimulus interval (ISI) of 4 s and a Poisson-distributed ISI length). All stimuli were on the screen for exactly 3 s. ISI was never less than 2 s or more than 7 s. This design ensured that the affective quality remained effectively constant within each block. Item modality was manipulated orthogonally to affect (i.e., all modalities are present at every affective level; Fig. 2B, top panel).

The twelve localized clusters of items (3 positive, 3 negative, 3 ambiguously valenced, 3 affectively neutral; Fig. 3a) were pseudorandomly presented across the functional EPI scans (four 49-s blocks per run with 18 s of fixation between each block; Fig. 3, bottom panel), such that each run contained one neutral block, one positive block, one negative block, and one ambiguously-valenced block. The ordering of these blocks was randomized within run. The items within each block were presented according to a fixed pseudorandomized order. Each run began with 9 s of fixation. All twelve clusters (containing a total of seventy-two items; 24 faces, 24 scenes, 24 sentences) were presented once in the first three runs, and then all items were repeated once again across the final three runs (6 runs total), but in a different order. A small white square was presented at the onset of each block (for 2 s) and a small black square was presented at the offset of each block (for 2 s) (these squares were modeled as regressors of no interest). Participants were asked to press a button when they saw the black square to ensure attention, and this task was successfully accomplished by all included participants. Other than this button pressing task, the task was a passive viewing design, and participants were simply instructed to look at the stimuli and keep their eyes open.

### Post-Scan Task

Following the scanner session, all participants completed a computer task in the lab where they provided affective ratings of the items that were presented in the scanner. Items were rated for arousal using a 9-point Likert scale and valence using a 3-alternative forced-choice task that consisted of the options “positive,” “negative,” and “no emotion.” Post-scan procedures were identical to those used in previous work, which has shown that these two rating responses can be mathematically combined to generate continuous values along the dimensions of positivity, negativity, and linear valence (Mattek et al., 2017), allowing the items to be effectively mapped into either theoretical space under consideration here. These behavioral data verified the assignment of each item into their respective affective conditions. The ratings of subjects in our task matched the data from other samples that was used to construct Fig. 3a. In other words, the subjects gave ratings in close agreement with the intended categorization of the stimuli.

### Operationalization of Valence, Arousal, Positivity, and Negativity

Subjective ratings in response to the experimental stimuli served as our operationalization of valence and arousal. Valence was rated using a 3-alternative forced-choice task, where participants classified a stimulus as either positive, negative, or neutral. From these data, a probability of each class was estimated for each stimulus. Arousal was rated using a 9-point Likert scale anchored at “not at all intense” to “very intense.” From these data, the average rating was estimated for each stimulus. Valence, positivity, and negativity were computed from these data, using the equations established in Mattek et al., 2017.

### Data Analysis

All fMRI data were preprocessed using a standard pipeline of functions in AFNI (Cox, 1996), which included: slice time correction, registration of all EPI images to the first EPI image, alignment of anatomical and EPI images, alignment to a standard anatomical space (Montreal Neurological Institute [MNI]-152 space) using AFNI’s @auto_tlrc function; smoothing with a Gaussian kernel of 6 mm; and normalizing the signal to a mean of 100 such that beta weights would reflect percent signal change. Each participant’s data was modeled using a general linear (GLM) approach with AFNI’s 3dDeconvolve function, which models the BOLD signal time course of each voxel using an array of linear regressors (often referred to as the design matrix). For this design, there were 3 “state” regressors and 21 “item” regressors. The state regressors modeled the affective quality of the stimulus blocks: one regressor modeled the stimulus blocks in general (blocks of stimulus-on versus fixation) and two regressors parametrically modulated this general on/off block regressor according to the affective quality of the items within each block. These two modulating regressors capture the effects of the affective manipulation, which are the primary regressors of interest in this paper. These two regressors are the only thing that changes between the Model Valence Arousal analysis (valence and arousal) and the Model Positivity Negativity analysis (positivity and negativity), which is described in more detail in the following paragraph.

For Model Valence Arousal, the 2 modulating state regressors were defined with an arousal value and a linear valence value, respectively, which was effectively constant across each block (by design) and reflected the affective quality of the cluster of items presented in that block. These modulating values were estimated using the post-scan session rating data, by averaging across all participants and all items within each block. On the other hand, for Model Positivity Negativity (Fig. 1b), the 2 modulating state regressors reflected the positivity value and the negativity value, respectively, which was also effectively constant across each block (by design) and reflected the affective quality of the cluster of items presented in that block. These values were also estimated using the post-scan session rating data. Note that positivity and negativity are difficult to model as independent regressors, because in practice, they are usually inversely correlated variables, which was also true in this experiment. However, the inclusion of the ambiguously-valenced condition allowed us circumvent this issue and impose perfect orthogonality between these regressors: for the clearly positive blocks, the unipolar positivity modulating regressor was set to 1 and the unipolar negativity modulating regressor was set to 0; for the clearly negative blocks, the unipolar positivity modulating regressor was set to 0 and the unipolar negativity modulating regressor was set to 1; for the ambiguously-valenced blocks, both modulating regressors were set to 1; and for the neutral blocks, both modulating regressors were set to 0 (note that these values were scaled to appropriately sum to zero, of course). This approach ensured that the positivity and negativity regressors had a temporal correlation of exactly zero. Mathematically, inclusion of all four conditions is required to achieve this orthogonality.

All other regressors remained fixed across both Models Valence Arousal and Positivity Negativity. The 21 item regressors captured the presence of the 3 particular stimulus modalities in this design (faces, scenes, sentences), allowing for estimates of 7 hemodynamic-response time points for each modality. The area under the estimated hemodynamic-response curve was used for further analyses of the item modality effects at the group level. Finally, 23 regressors of no interest were as follows: 7 hemodynamic-response time points for the start cues at the beginning of each block (2-s white square) and 7 hemodynamic-response time points for stop cues at the end of each block (2-s black square), 6 motion regressors, and 3 polynomial regressors to account for scanner drift (zero-, first-, and second-order).

These models were applied to each individual subject, and the resulting beta weights for each voxel were then carried over to a group analysis, to see which voxels were linearly related to the affective dimensions at the group level. Functional regions associated with each affective dimension were selected using a reasonable statistical threshold (false discovery rate set to 0.05, cluster size > 20 contiguous 2.5 × 2.5 × 2.5 mm voxels).

## Results

The primary effects of interest for this report are related to the different affective qualities that form the experimental conditions. As described in the previous section, affective quality was modeled in two ways: first by imposing contrasts along the valence and arousal dimensions (i.e., Model Valence Arousal, contrasts a and b described in “Methods”); second by imposing contrasts along the positivity and negativity dimensions (i.e., Model Positivity Negativity, contrasts c and d described in “Methods”). Here, we compare the results yielded by each pair of contrasts, with particular attention to the theoretical predictions described in the first part of the “Methods” section.

### Effects of Affective Quality: Quantitative Comparison of Models Using Whole-Brain Analysis

**Fig. 4:**
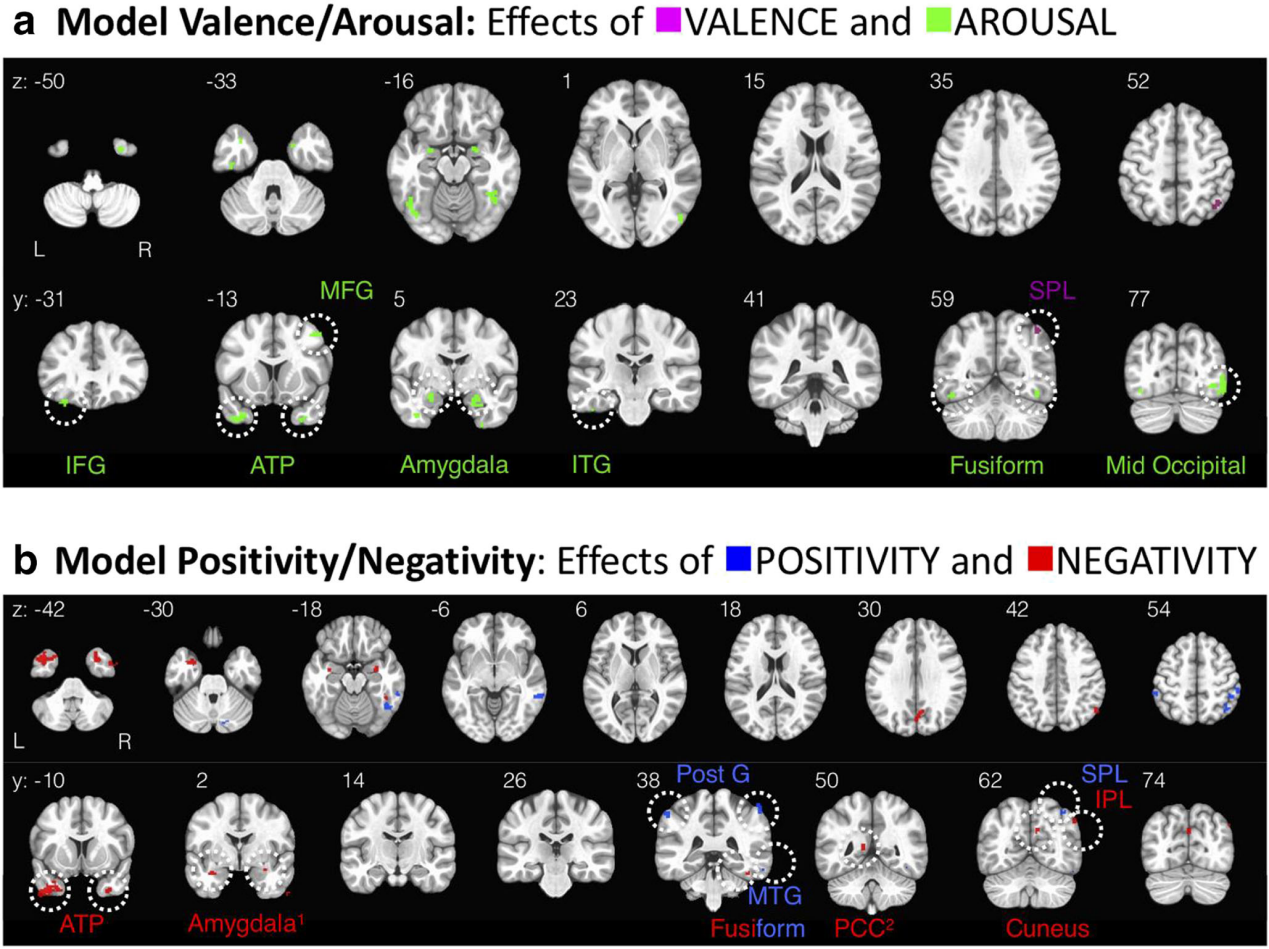
Maps of functional brain regions that show statistical effects linearly related to each affective dimension: **a** Model Valence Arousal: voxels tracking linear valence are magenta and voxels tracking arousal are green; **b** Model Positivity Negativity: voxels tracking positivity are blue and voxels tracking negativity are red. Note that all (expect one^2^) of these functional regions have positive beta weights, and colors reflect the affective dimension the region is functionally related to, not the degree or direction of relationship. Regions yielded by Model Valence Arousal and regions yielded by Model Positivity Negativity are almost entirely non-overlapping (this is why the depicted slices are not the same for each model, see all instances of overlap in Figs. 5, 6, and 7). These clusters (all > 20 contiguous, 2.5 × 2.5 × 2.5 mm voxels^1^) survived a reasonably conservative correction that set the false discovery rate to 0.05. ATP (anterior temporal pole), IFG (inferior frontal gyrus), IPL (inferior parietal lobule), ITG (inferior temporal gyrus), MFG (middle frontal gyrus), MTG (middle temporal gyrus), PCC (posterior cingulate cortex), Post G (postcentral gyrus), SPL (superior parietal lobule). ^1^Cluster size note: The cluster size threshold of 20 voxels was chosen prior to viewing the results. However, the negativity clusters for amygdala were included in this map even though they are slightly smaller in size (left: 16 contiguous voxels and right: 19 contiguous voxels) than the threshold set for the entire brain (all other regions > 20 contiguous voxels), due to the general relevance of the amygdala as a region of interest in the field of affective neuroscience, we thought was important to note these clusters. The arousal-sensitive clusters within amygdala shown in (**a**), were > 20 voxels, consistent with the whole-brain threshold chosen prior to viewing the results. ^2^Statistical note: The PCC region is negatively associated with negativity (i.e., has a negative mean beta weight, all other regions depicted have positive mean beta weights)

Table 2 and Fig. 4 summarize the brain regions that track differences in affective quality when (a) linear valence and arousal are used to model differences in affective quality (Model Valence Arousal) or (b) positivity and negativity are used to model differences in affective quality (Model Positivity Negativity). These functional brain regions represent clusters of voxels (size > 25 contiguous 2.5 × 2.5 × 2.5 mm voxels) that survived a reasonably conservative statistical threshold (false discovery rate = 0.05; e.g., Bennett, Baird, Miller, & Wolford, 2009) for determining whether any given voxel’s activity, over time, was linearly related to the manipulation along a particular affective dimension while controlling for false positives.

**Table 2:** Functional brain regions showing significant activity related to Model Arousal/Valence and Model Positivity Negativity

	#Voxels	Peak MNI coordinate		
		*x*	*y*	*z*
**Arousal**	**733**			
Frontal lobe				
Left inferior frontal gyrus	33	52.5	− 30.8	− 12
	21	32.5	− 33.2	− 27
Right medial frontal gyrus	25	− 50	− 18.2	48
Temporal lobe				
Left anterior temporal pole	168	52.5	1.8	− 47
Right anterior temporal pole	79	− 30	4.2	− 49.5
Left amygdala	74	− 20	− 0.8	− 17
Right amygdala	21	22.5	1.8	− 17
Left inferior temporal gyrus	30	42.5	16.8	− 39.5
Left fusiform gyrus	57	45	71.8	− 19.5
Right fusiform gyrus	41	− 45	56.8	− 19.5
Occipital lobe				
Left middle occiptial gyrus	40	25	109.2	5.5
	26	52.5	86.8	8
Right middle occipital gyrus	118	− 47.5	79.2	− 14.5
**Valence**	**33**			
Parietal lobe				
Right superior parietal lobule	33	− 45	64.2	58
**Positivity**	**225**			
Temporal lobe				
Right middle temporal gyrus	54	− 70	39.2	− 9.5
Right fusiform gyrus	39	− 52.5	56.8	− 22
Parietal lobe				
Left postcentral gyrus	22	57.5	39.2	53
Right postcentral gyrus	67	− 57.5	34.2	63
Right superior parietal lobule	21	− 40	56.8	58
Non-cortical				
Right cerebellum	22	− 20	76.8	− 29.5
**Negativity**	**420**			
Temporal lobe				
Left anterior temporal pole	172	52.5	−10.8	− 47
Right anterior temporal pole	74	− 57.5	−0.8	− 47
Left amygdala	19	32.5	4.2	− 19.5
Right amygdala	16	− 30	4.2	− 17
Right fusiform gyrus	38	− 42.5	34.2	− 27
Parietal lobe				
Right cuneus	42	0	71.8	30.5
Right angular gyrus	32	− 50	74.2	38
Left posterior cingulate gyrus	27	5	56.8	15.5

A major takeaway from the whole-brain results is that each of the two psychological models (Model Valence Arousal versus Model Positivity Negativity) reveals a substantially different answer to the question of *where* affective information is represented in the brain, as the functional regions yielded by each model are essentially non-overlapping. This observation in and of itself supports the notion that all four dimensions (arousal, valence, positivity, negativity) are partially independent, rather than a rigid linear rotation of each other, in support of the new theoretical synthesis about the underlying dimensional structure (Mattek et al., 2017). It is additionally important to note that the set of functional regions associated with each model fit together like puzzle pieces in particular regions of interest, in a striking way that cannot be readily ascribed to chance, further supporting the legitimacy of the new approach of looking at all four dimensions separately. The effects in these regions of interest are described in more detail in the following section.

### Effects of Affective Quality: Qualitative Comparison of Models Using Whole-Brain Analysis

If we consider the union of the functional brain regions yielded by Model Valence Arousal and Model Positivity Negativity, some strikingly organized patterns emerge in the data. For simplicity, we highlight the three regions which show effects along more than one psychological dimension: fusiform gyrus, amygdala, and anterior temporal pole (ATP), as well as the only region that tracked linear valence: right SPL. The anatomical arrangements of activations in each of these regions is described in more detail below.

#### Fusiform Gyrus

**Fig. 5:**
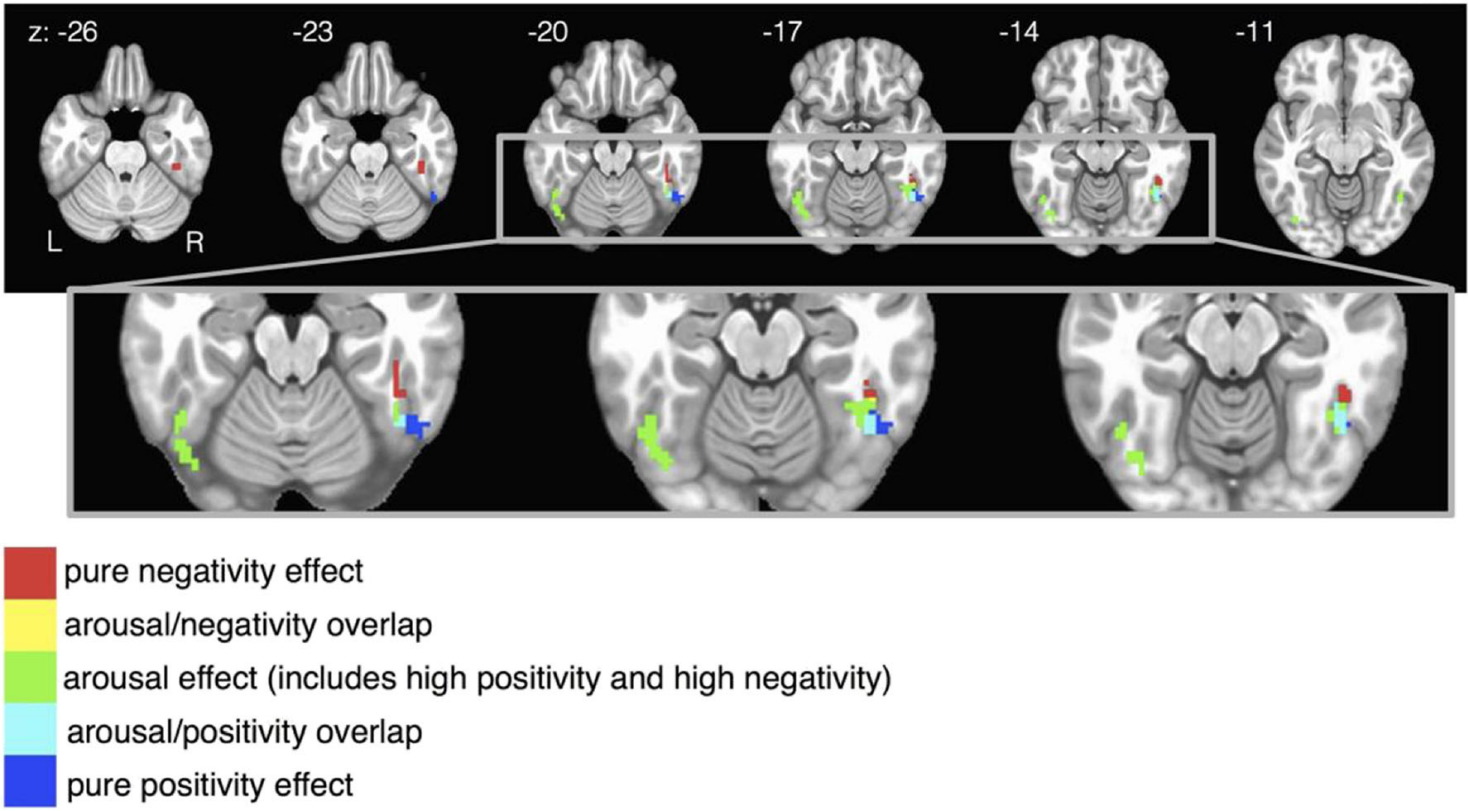
Fusiform gyrus results from Fig. 4 a and b are combined and focused on in this figure. In the right fusiform, valence is represented along an anatomical gradient. Purely negative-sensitive voxels are more anterior, purely positive-sensitive voxels are more posterior, and arousal-sensitive voxels (which show increased activity to positivity and/or negativity compared with affectively neutral stimuli) are anatomically interposed between the pure valence regions. Combining the results from both theoretical models Valence Arousal and Positivity Negativity are necessary to see this pattern. This pattern would have been overlooked if only one of these models had been selected a priori. Colors represent affective condition, not beta weights: all beta weights are positive and survived a reasonably conservative statistical threshold that set the false discovery rate to 0.05

The anatomical arrangement of activations in bilateral fusiform gyrus is shown in Fig. 5. Activity in the fusiform gyrus tracks the arousal dimension bilaterally, but in the right hemisphere, this gyrus also tracks positivity and negativity. That is, the right fusiform represents each unipolar valence dimension (positivity, negativity), but not linear valence. Strikingly, the sub-regions within right fusiform that are sensitive to negativity, arousal, and positivity, respectively, are neatly anatomically organized from the more anterior aspects to the more posterior aspects of the gyrus, revealing a right-lateralized gradient representation of valence in the fusiform, which is situated just dorsally to the fusiform face area (FFA, see section on “Effects of Item Modality: Connection to Effects of Affective Quality”).

#### Amygdala

**Fig. 6:**
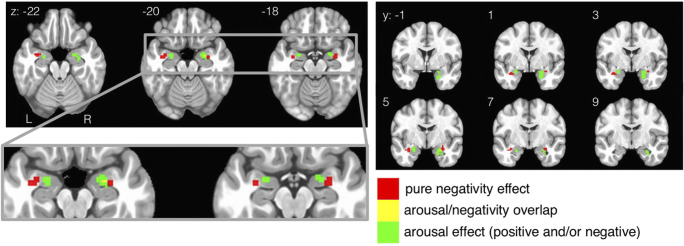
Amygdala results from Fig. 4 a and b are combined and focused on in this figure. Responses related to negativity and arousal are largely non-overlapping (except for 2 voxels). Negativity is represented more laterally whereas arousal is represented more medially. Combining the results from both theoretical models Valence Arousal and Positivity Negativity are necessary to see this pattern, which would have been overlooked had either model alone had been selected a priori. Colors represent affective condition, not beta weights

The spatial arrangement of activations in the amygdala is shown in Fig. 6. Both negativity and arousal are represented within the amygdala, but in distinct locations. Voxels sensitive to negativity are located in the lateral aspects of bilateral amygdala, whereas voxels sensitive to arousal are located more dorsal-medially. Had Model Arousal/Valence been chosen a priori, the resulting conclusion for this dataset would have been that the amygdala tracks arousal generally (i.e., positive and/or negative conditions). Had Model Positivity Negativity been chosen a priori, the resulting conclusion for this dataset would have been that the amygdala tracks negativity but not positivity. Only by acknowledging the synthesis of both dimensional structures can we see that the amygdala tracks both arousal and negativity in distinct anatomical locations.

In turn, these data shed light on an existing debate about whether the amygdala represents information about valence or arousal (e.g., Jin et al., 2015; Kim et al., 2017). In many cases, the amygdala is found to be specifically sensitive to negative valence (e.g., LeDoux, 1998; Öhman, 2005), but other experiments show it is also sensitive to positivity/reward (e.g., Garavan, Pendergrass, Ross, Stein, & Risinger, 2001; Kensinger & Schacter, 2006; Douglass et al., 2017). In the current data, Model Valence Arousal shows the amygdala tracking general arousal (and not linear valence), which would suggest the amygdala is sensitive to both positivity and negativity. However, Model Positivity Negativity shows the amygdala tracking negativity but not positivity), suggesting that this structure has a bias for processing negative information. By examining the data with both models, we can see that the amygdala represents information about both valence and arousal, rather than being exclusively dedicated to processing a particular dimension.

Our results within bilateral amygdala show that negativity is represented in lateral amygdala whereas arousal is represented more dorsal-medially. This anatomical arrangement of activity can be interpreted based on known signal flow through this anatomical structure: inputs from the visual ventral stream come in laterally (i.e., the basal-lateral nucleus; Aggleton, 1992) and output to the hypothalamus and brainstem exit dorsal-medially in humans (i.e., the central nucleus; Whalen & Phelps, 2009). With this signal processing pipeline in mind, perhaps the amygdala is able to transform a negativity input signal received laterally into a general arousal signal at the output nuclei, which might receive inputs about positivity from some other source. However, although the central nucleus is generally known as an output that coordinates emotional responses, some signals flow through this structure in the opposite direction (e.g., Yu, Ahrens, Zhang, Schiff, Ramakrishnan, Fenno et al., 2017). Future work would be needed to fully address the directionality of signal flow in this task.

#### Anterior Temporal Pole

**Fig. 7:**
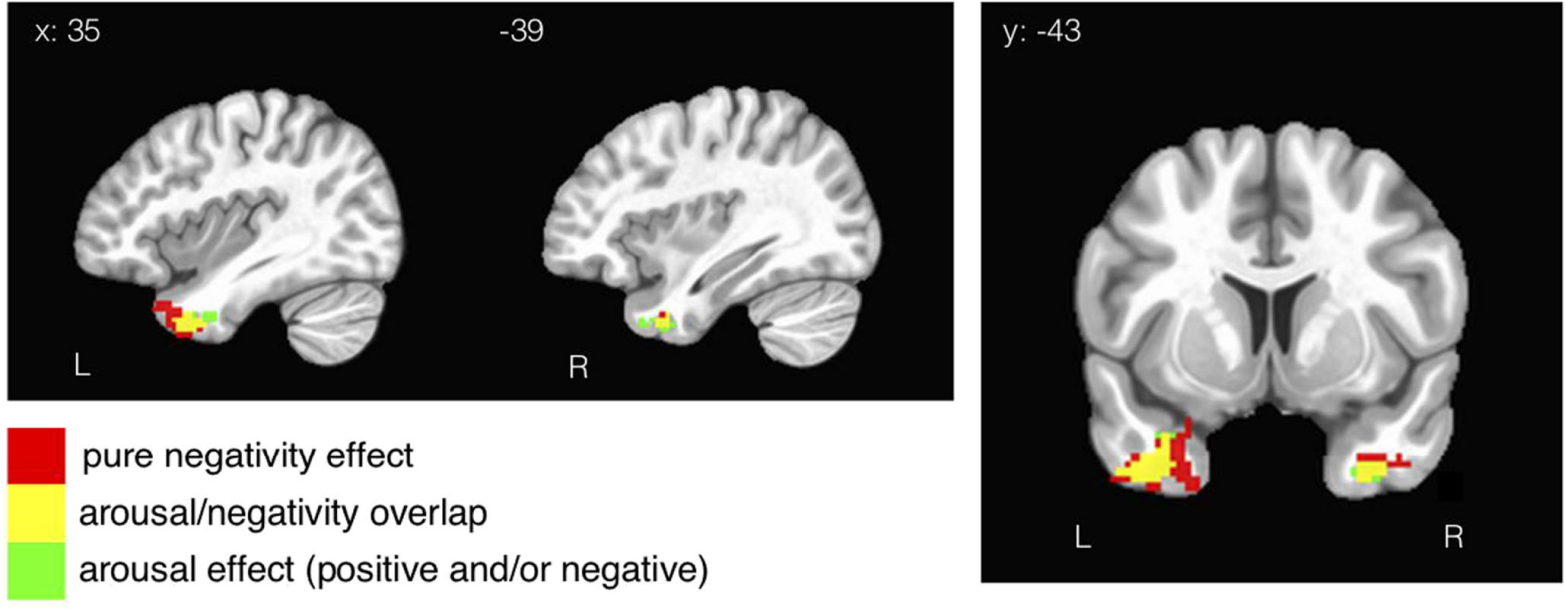
ATL results from Fig. 4 a and b are highlighted here to give the reader a better illustration of the overlapping effects in this region. ATL was the only region that exhibited substantial overlap across Model Arousal/Valence and Model Positivity Negativity

Like the amygdala, the ATP tracks both negativity and arousal. However, unlike the patterns in the fusiform and amygdala, representations of unipolar negativity and arousal have substantial overlap in ATP (Fig. 7). Interestingly, this is the only brain region where there is substantial overlap across any of the psychological dimensions in this design (fusiform and amygdala have a small overlap between regions but are still mostly distinct, as shown in Figs. 5 and 6).

#### Superior Parietal Lobule

SPL is the only brain region whose activity was linearly related to the valence dimension. In the rest of the brain, the representation of valence is specific to either positivity or negativity. Here, the effect was found in the right hemisphere specifically. Had Model Positivity Negativity been assumed a priori, this effect of linear valence would have remained concealed.

The SPL is the only functional region that our experiment identified as being linearly related to valence. It is worth noting that the superior parietal lobule has been implicated in the general representation of quantitative number lines (Dehaene, Piazza, Pinel, & Cohen, 2003). This suggests that this region also represents affective quality along a number line, such that the activity is greater for more positive information and lower for more negative information. This result frames the perception of affective quality as a form of magnitude calculation, consistent with existing work showing SPL represents more general forms of magnitude, including the magnitude of physical, temporal, and social distances (Parkinson, Liu, & Wheatley, 2014).

### Effects of Item Modality: Connection to Effects of Affective Quality

Due to the structure of the experimental design, it was possible to separate out the effects of looking at a particular type of image (face versus complex scene versus short sentence), because item modality was manipulated orthogonally to the affective quality of the images. For example, by extracting regions sensitive to the presentation of faces versus other modalities, we were able to estimate the location of the FFA at the group level and determine that the FFA was located just ventral to the region representing valence and arousal in the bilateral fusiform gyrus (see section above on Fusiform results). In other words, the gradient representation of valence in the right fusiform seems to be built on the dorsal edge of the functional region dedicated to a more general representation of faces.

The functional regions that track the presence of any given stimulus modality in this experiment (i.e., faces versus complex scenes versus sentences) are anatomically tied to the regions that track affective quality. For example, bilateral SPL showed increased activity to the presence of sentences, and an adjacent SPL region on the right was proportional to changes in linear valence. Further, a region of the cerebellum that activated to the sentence modality has an adjacent cerebellar region that was proportional to changes in positivity. Complex scenes (e.g., IAPS) evoked activity across large portions of the dorsal and ventral visual streams, with the ventral activity extending anteriorly all the way to the amygdala, a structure that was implicated in the processing of affective quality in this experiment and more generally. Finally, the regions tracking the arousal dimension (Table 1, Fig. 3a) generally correspond to known regions that appear in face localizers (e.g., fusiform gyrus, middle occipital gyrus, anterior temporal pole; Haxby, Hoffman, & Gobbini, 2000).

### Traditional ROI Analysis

We conducted more traditional ROI analyses in order to test whether or not the beta weights yielded by the four affective contrasts (bipolar valence, arousal, positivity, negativity), averaged within ROI, were significantly different from each other. Given that the effects of affective quality seemed to align somewhat with the effects of item category (e.g., image versus sentence versus face), we used the item contrasts to generate ROIs that were independent of affective quality in the anterior temporal lobe (ATL) and the amygdala using the image versus other items contrast; the superior parietal lobe (SPL) using the sentences versus other items contrast; and the fusiform face area (FFA) using the face versus other items contrast. We then extracted the average beta weights for the affective quality regressors from these ROIs, resulting in one beta weight for each subject for each ROI. We then performed one-way ANOVAs for each ROI, to see if there was an effect of affective dimension. The effect of affective dimension in the ATL was significant in both hemispheres, such the effect of negativity was greater than the other conditions (left ATL: *F*(3, 24) = 3.149, *p* = 0.030; right ATL: *F*(3, 24) = 4.641, *p* = 0.005). The amygdala ROI showed the same general configuration as the ATL (negativity greater than other conditions), but the differences were not significant (left amygdala: *F*(3, 24) = 2.407, *p* = 0.075; right amygdala: *F*(3, 24) = 2.383, *p* = 0.077). The ANOVAs for SPL was significant only on the right side, consistent with the valence effect in Table 2 (left SPL: *F*(3, 24) = 0.742, *p* = 0.531; right SPL: *F*(3, 24) = 3.242, *p* = 0.027). The FFA effects were not significant (left FFA: *F*(3, 24) = 0.291, *p* = 0.832; right FFA: *F*(3, 24) = 0.54, *p* = 0.657). These results suggest that the effects of affective quality do not lie exactly within the regions that are sensitive to item category, but are rather in more specific functional regions yielded by the whole-brain analysis. For example, the regions in the fusiform gyrus that were significantly related to different affective dimensions (in Fig. 5) in the whole-brain analyses were slightly dorsal to the faces versus items contrast.

## Discussion

In this paper, we compare two theoretical models that are routinely used to analyze affective quality. One model uses the dimensions of valence and arousal, and the other model uses the dimensions of positivity and negativity. Neither model was obviously superior to the other in terms of explaining the data in this experiment, as each model produced a similar number of voxels with significant effects.

However, qualitatively, we find that the spatial arrangement of functional brain regions associated with each established model are largely non-overlapping. Therefore, rather than showing one model to be superior to the other, these data showed that both models are useful, but they each capture different parts of the data (different regions of the brain). This means that some brain activity was best modeled by the valence or arousal dimensions, whereas other brain activity was best modeled by the positivity and negativity dimensions.

These results challenge commonly employed theoretical assumptions in the field (outlined in the introduction and experimental predictions), which assume that one pair of these dimensions is more optimal than the other, with the practical consequence being that researchers routinely only choose a pair of these dimensions rather than employing all four. For example, we observe that effects of positivity and effects of negativity are in non-overlapping anatomical locations, which runs contrary to the theoretical assumption that positivity and negativity have a purely inverse linear relationship and should be analyzed as a bipolar phenomenon (Green, Goldman, & Salovey, 1993; Russell & Carroll, 1999; Russell, 2017). Rather, the current data suggest that much brain activity is best modeled with two unipolar dimensions, even though some regions of the brain (the SPL in particular) was best modeled with bipolar valence.

### Limitations

The present study is not without limitations that could be addressed in future investigations. First, the present findings rely on univariate analyses of fMRI data, which are relatively less sensitive than multivoxel pattern analysis (MVPA) in general. Subsequent studies employing MVPA may be able to uncover specific neural activation patterns associated with affective quality that were not previously observable through a univariate approach. Second, as the present study is designed to test existing theoretical frameworks of emotion in the context of fMRI research, our data are not suited to discover new dimensions of affective quality. Some new studies find additional dimensions (e.g., Cowen, Fang, Sauter, & Keltner, 2020), and other new studies continue to confirm valence and arousal as principle dimensions (Jackson, Watts, Henry, List, Forkel, Mucha et al., 2019). Future studies involving a vast dataset that surveys affective quality from a broad range of stimuli may be able to utilize a data-driven approach to meet such goals. Third, our main findings showing a spatial gradient of functional activations should be carefully interpreted, given the inherent limitations associated with the spatial resolution of fMRI data.

Finally, the nature of our passive viewing task also limits the interpretation of the current data. Affective dimensions such as positivity and negativity are thought to be systems of motivation (e.g., Cacioppo & Berntson, 1994; Lang, 1995) that have evolved to guide behavior. Therefore, the neural systems should relate to organized action and physiology. Understanding the behavioral outcomes related to the neural findings outlined here will require future work.

### Generalizability

The generalizability of the patterns observed here are likely constrained by task parameters, specifically, item modality. The regions tracking affective quality seem to be anatomically close to, but distinct from, functional regions tracking item modality. This would suggest more broadly that representations of affective quality will change anatomical location in the brain, depending on relevant sensory modalities or other task parameters. It follows that meta-analyses that combine experiments employing different item modalities to evoke affective quality are at risk for averaging out real effects of that are specific and predictable based on non-affective task parameters.

Along these lines, one would predict that the valence gradient seen around FFA in this design would perhaps appear in a region other than the fusiform gyrus, if the task did not prominently feature faces as a stimulus modality. Indeed, valence gradients have been identified in other brain regions in the rodent literature, namely the nucleus accumbens shell has a rostrocaudal valence gradient that codes for the approach/avoid properties of habitual behaviors (Reynolds & Berridge, 2002; Reynolds & Berridge, 2008), and a mirrored valence gradient in the prefrontal cortex can selectively bias or inhibit the expression of valenced behaviors through projections to the nucleus accumbens shell (Richard & Berridge, 2013). In this sense, valence gradients might be a more fundamental organizing principle that manifests in many different brain networks.

### Overall Summary

To help understand what we can conclude from these results, consider an analogy in which the variable of physical temperature (hot versus cold) takes the place of the variable of psychological valence (positive versus negative). Consider how your own bodily response to temperature varies around an equilibrium point, such that there is a certain set of physiological processes that are engaged when the system is too cold and a quite different set of processes that are engaged when the system is too hot. Any physiological feature of these processes can be observed and measured following a controlled manipulation of temperature, and these features are not necessarily quantitatively opposite in their structure. That is, when it is cold, there might be the occurrence of “goosebumps” which pulls the skin out, but there is no literal inverse of goosebumps that pushes the skin inward causing dimples when it is hot. With this example, it is easy to see that it would be an error to model the textural properties of the skin as a linear response to temperature. Here, we show that the structure of the biological response to valence manipulations (as measured by fMRI) have the same inherent structure as the biological response to temperature manipulations, such that responses to opposite ends of the dimension are not opposite in their measurable form. This general pattern has been demonstrated with other physiological measurements, not just fMRI (e.g., see Lang, 1995 for a review).

To take this analogy further, consider that the equilibrium point for subjectively felt temperature is a point of optimization that, by definition, minimizes the amount of metabolism that needs to be dedicated to regulating the temperature of the system. In turn, a sufficient change in temperature away from equilibrium, in either direction, will cause physiological changes associated with a general metabolic increase (like sweating, which occurs in both hot and cold states). Here, increases along the arousal dimension are analogous to the general metabolic increases required for temperature regulation as the system moves away from the equilibrium point, regardless of direction.

Still, despite this non-linear pattern of responses to hot versus cold temperature, our conception of temperature as a linear dimension is not an error. We can readily point out naturally occurring features that vary linearly with temperature (such as the density of liquid or the speed of sound). Furthermore, we can subjectively feel the gradient of temperature as it changes, for example, when we turn the heat on in a cold room, if we overshoot we can feel the transition from feeling cold to feeling hot happen over time. Although it is possible, it is relatively unusual for part of the body to be hot and for part of it to be cold simultaneously, so the presence of one state tends to exclude the other. Using the logic of this analogy, we can see how it is correct to simultaneously acknowledge both the opposition of positivity and negativity (linear valence) as well as the independence of the biological response patterns to positivity versus negativity.

To summarize, valence and arousal are important psychological variables that influence a wide range of neuropsychological processes, such as attention, memory, and decision-making. This paper demonstrates a technique for designing experiments and/or modeling manipulations that captures the effects along each of these affective dimensions. The method demonstrated here is based on theoretical principles that are aligned with observed behavior (Mattek et al., 2017). We apply the method in conjunction with fMRI measurements, which yields insights about how affective quality is represented by the brain. These insights would have remained concealed had commonly used two-dimensional approaches been employed. Most generally, this paper offers a proof-of-concept as to how organizing variables at the level of psychological theory can enhance the interpretation of biological measurements.

## Additional Information

## Data Availability

The language in the IRB-approved consent forms signed by participants does not allow the authors to make these data publicly available. However, data can be made available by contacting the corresponding author.

## Footnotes

1 The logic here could be applied to any dependent measure.

2 Another way of potentially describing this partial redundancy is to say that the system of variables has a fractional dimensionality between 2 and 3.

3 Five participants were excluded for excessive motion, and one for presumably falling asleep, which was inferred from a lack of ventral visual stream activity that was robustly present for every other participant in this visual task. Excessive motion during functional scans was indicated by biologically implausible spikes (> 1.5 mm movement occurring in > 10% TRs) in the signal. *All exclusions were decided prior to group analyses*.
